# Synthesis and *in Vitro* Cytotoxic Activity of Compounds with Pro-Apoptotic Potential

**DOI:** 10.3390/molecules15010012

**Published:** 2009-12-24

**Authors:** Giselle Apicela Soares, Renata Barbosa de Oliveira, Saulo Fernandes de Andrade, Ricardo José Alves, Carlos Leomar Zani, Elaine Maria de Souza-Fagundes

**Affiliations:** 1 Laboratório de Química de Produtos Naturais, Centro de Pesquisas René Rachou, Fundação Oswaldo Cruz (FIOCRUZ), Av. Augusto de Lima 1715, Belo Horizonte, MG, 30190-002, Brazil; 2 Departamento de Produtos Farmacêuticos, Faculdade de Farmácia, Universidade Federal de Minas Gerais (UFMG), Avenida Antônio Carlos 6627, Belo Horizonte, MG, 31.270-901, Brazil; 3 Departamento de Fisiologia e Biofísica, Instituto de Ciências Biológicas, Universidade Federal de Minas Gerais (UFMG), Avenida Antônio Carlos 6627, Belo Horizonte, MG, 31.270-901, Brazil

**Keywords:** carbohydrates, furan derivatives, cytotoxic activity, anti-tumor, apoptosis

## Abstract

In our search for new anticancer therapies, some compounds synthesized in our lab were selected and their potential cytotoxic activity was evaluated *in vitro* against two cancer cells lines including a solid tumor (UACC-62, melanoma) and a human lymphoma (JURKAT). Compounds showing cytotoxic activity were subjected to an apoptosis assay. Two compounds showed promising results.

## Introduction

The progressive elucidation of molecular mechanisms involved in cancer has opened up a new horizon for the development of new antitumoral compounds [1]. Specifically, for profiling the activity of anticancer drugs, the major indicators of these agents include inhibition of proliferation and induction of apoptosis in cancer cells [2]. Thus, direct manipulation of the biochemical machinery that regulates apoptosis is an interesting and new therapeutic approach to cancer [3].

Apoptosis or programmed cell death not only plays a crucial role in tissue development and homeostasis, but is also involved in a range of pathological conditions and now recognized as an important component of multi-step carcinogenesis and therapy resistance [1,4]. This physiological phenomenon represents the terminal morphological and biochemical events [5] and occurs through the activation of a cell-intrinsic suicide program. This program is carried out by internal, as well as external signals, divided in various phases terminating with signals that initiate the process leading to cellular destruction, characterized by DNA fragmentation as well as by loss of mitochondrial membrane integrity and liberation of molecules that initiate intracellular proteases activation [6]. At the center of the apoptosis machinery is a family of intracellular proteases, known as 'caspases', that are responsible directly or indirectly for the morphological and biochemical events that characterize classical apoptosis [7].

Apoptotic pathways might be significantly altered in cancer cells with respect to normal cells, and these differences might present a therapeutic window that can be exploited for the development of useful anticancer drugs. Moreover, cancers that possess alterations in proteins involved in cell death signaling are often resistant to chemotherapy and are more difficult to treat using chemotherapeutic agents that primarily work by inducing apoptosis [8].

Several compounds prepared employing classical organic and combinatorial chemistry have advanced into clinical trials or are already approved. Research in the last decade has revealed a promising future for apoptosis based cancer therapies [9]. In this scenario of discovery of small molecule modulators of apoptosis, considerable effort is being aimed at improving the prototypic drugs and replacing them by small molecule organic compounds, which could set the stage for future therapeutics [10].

**Figure 1:**
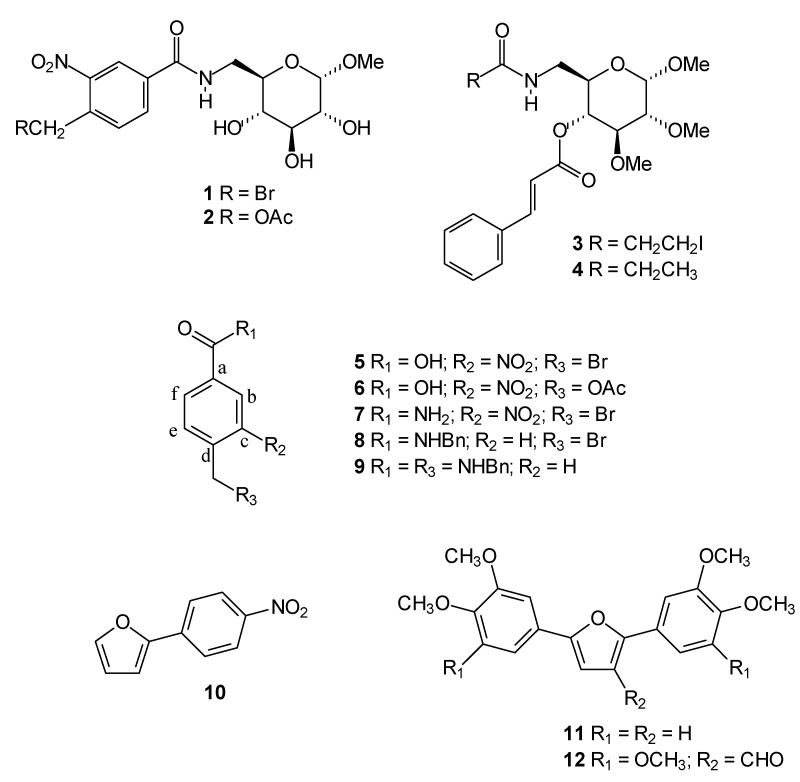
Chemical structures of compounds tested as cytotoxic agents.

With this in mind, we decided to evaluate the cytotoxic activity of twelve compounds previously synthesized in our laboratory (Figure 1) to potentially identify novel small-molecule compounds with potential anti-cancer properties. We performed this investigation using an *in vitro* bioassay based on their cytotoxic effects against cancer cells, including UACC-62 (human melanoma) and Jurkat (human leukemia T-cell line) measured by the MTT method. The active compounds were investigated to determine if they also act as apoptosis inducers as evaluated by quantification of subdiploid DNA content and caspase 3 activation by flow cytometry.

The carbohydrate derivatives **1**-**5** were chosen to evaluate their cytotoxicity because there is evidence that the activity of some compounds can be tuned by changes to the monosaccharide core [11]. Moreover, carbohydrates play crucial roles in many biological processes and, therefore, their presence can be important to modulate the cytotoxic activity.

The bromomethyl, acetoxymethyl and iodoethyl groups present in compounds **1**-**3, ** respectively**,** are particularly reactive and these compounds can act as alkylating agents. Binding of alkylating agents to cellular DNA is considered to be a lethal event associated with anticancer activity [12]. Compounds **5**-**8**, containing the alkylating groups but without the carbohydrate moiety, were included for comparison. Compound **4**, presenting the carbohydrate moiety but without the alkylating groups, was also included for comparison.

We have previously reported the trypanocidal activity of compound **9** [13]. Some studies have demonstrated a correlation between trypanocidal and antitumor activities [14]. Thus, we decided to investigate the effects of compound **9** on the growth of cancer cells. Recently, we also reported the evaluation of the cytotoxicity activity of arylfurans **10** and **11** against the human cancer cells lines MCF-7 (breast), TK-10 (renal) and UACC-62 (melanoma) [15], which motived us to perform a more detailed investigation of the ability of these compounds to induce apoptosis. Diarylfuran **12** was also selected based on its structural similarity to the diarylfuran **11**.

### Chemistry

**Scheme 1:**
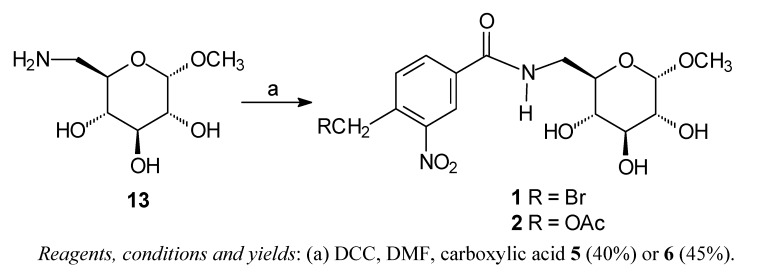
Synthesis of the amides **1** and **2**.

The carbohydrate derivatives **1** and **2** were prepared by treatment of methyl 6-amino-6-deoxy-α-D-glucopyranoside **13** [16] with carboxylic acid **5** or **6** and DCC (Scheme 1). The amido-esters **3** and **4** were synthesized from readily available methyl α-D-glucopyanoside in seven steps using methods previously reported by us [17].

The compounds **5** and **6** were prepared in two and three steps, respectively, from *p*-toluic acid, according to literature procedures [18,19,20]. The benzamide **7** was obtained from the direct reaction of carboxylic acid **5** and NH_4_Cl/SiO_2_, triethylamine (TEA) and tosyl chloride under solvent-free conditions [21]. Synthesis of the amides **8** and **9**, was carried out by reaction of the acyl chloride, obtained from 4-(bromomethyl)benzoic acid [19] by means of reflux with thionyl chloride, with benzylamine. Benzylamine was added either with an equimolar amount to obtain the amide **8** or in an excess to obtain the amide **9**.

**Scheme 2:**
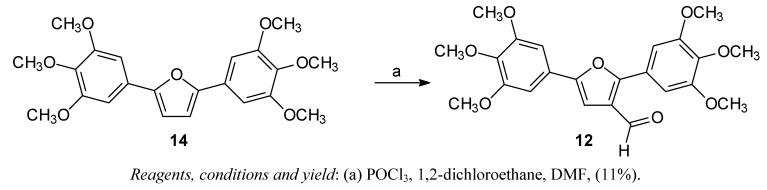
Synthesis of the diarylfuran **12**.

The arylfuran **10** was synthesized in one step using the classical Meerwein arylation (treatment of furan with diazonium salts in presence of cupric salts), as described in previous work [22]. The diarylfuran **11** and 2,5-bis-(3,4,5-trimethoxy)furan **14** were prepared in two steps via a Stille coupling reaction [22]. The diarylfuran **12** was obtained from **14** by Vilsmeier-Haack formylation (Scheme 2).

### Antiproliferative activity

Screening of synthesized substances was carried out using two human cancer cell lines: UACC-62 (human melanoma) and Jurkat (derived from human T-cell leukaemia). Proliferation percentage was determined by the modified 3-(4,5-dmethylthiazol-2-yl)-2,5-diphenyltetrazolium bromide (MTT) assay [23], based on the ability of a mitochondrial dehydrogenase enzyme from viable cells to cleave the tetrazolium rings of the pale yellow MTT and form dark blue formazan crystals. Both lineages were incubated with the test substances at 100 μM for 48 hours and the cell proliferation/viability determination using the survival percentage obtained with the cell treated only with the vehicle (0.1% aqueous DMSO) as reference. In all experiments, etoposide (20 μM) was the positive control of a reference chemotherapeutic used in clinic. The results are expressed as the average of triplicate assays.

### Analysis of DNA Fragmentation

In order to study the relationship between cell proliferation inhibition and the induction of apoptosis, we decided to study the subdiploid DNA contends as indicative of DNA fragmentation by apoptosis. We selected active cytotoxic substances to one or both cell lines and evaluated their pro-apoptotic potential. We used the method to detect apoptotic nuclei, as described by Nicoletti and colleagues [24]. This flow cytometric method is useful for measuring the percentage of apoptotic nuclei after propidium iodide staining in hypotonic buffer for assessing apoptosis. The cells were treated with 100 μM of compounds for 18 h and incubated for 4 h at 4 °C, and PI fluorescence of individual nuclei was measured by flow cytometry. The percentage of hypodiploid nuclei correlates with the extent of apoptosis in the samples. The results represent the average ± SD in triplicate samples. Every experiment was repeated at least three times. With regard to apoptosis induction, a result is considered positive when the obtained level of DNA fragmentation at least doubles the values obtained for the control cultures that were treated only with the solvent.

#### Caspase 3 Activation

We evaluated if the DNA fragmentation was connected with caspase-activation dependence since various anticancer drugs have been reported to induce caspase-3 activation leading to apoptosis. Therefore, we examined involvement of the principal executing caspases - caspase-3, which clearly emerged as the single most important cysteine protease during the execution phase of apoptosis. The percentage of caspase-3 dimerized (actived) was determined by a single staining with a Caspase 3-FITC Antibody from BD Biosciences to detect the quantity of the apoptotic cells. This assay permits the confirmation of involvement of this enzyme in the cell death process; that is considered to be preliminary data for the determination of the mechanism of action.

## Results and Discussion

The effects of compounds **1**-**12** on the growth and viability of UACC-62 and Jurkat cells were investigated and the results are summarized in Table 1. The results are given in percent of cell growth compared to the untreated control cells (DMSO 0.1%).

Among the twelve compounds tested, four (**5**, **8**, **11** and **12**) displayed anti-proliferation effects (proliferation less than 60%) against one or both of the cancer cells lines. Compound **5** was found to be more toxic to the Jurkat cell line than to UACC-62. The results revealed the interesting effect presented by compound **11**, which exhibited cytotoxicity only against UACC-62 cells. In contrast, compounds **8** and **12** presented cytotoxic activity against both cells lines. The cytotoxicity of compounds **5** and **8** might be associated to their intrinsic alkylating properties. However, compound **1** and **7**, bearing benzylic bromine substituent as **5** and **8**, were inactive. The low cytotoxic activity of the compounds **1** and **7** may be related to their inadequate physicochemical properties and inability to cross cells membranes. The mechanism of action of diarylfurans **11** and **12** has not yet been proposed.

**Table 1:** Effects of compounds synthesized on the growth of human cancer cells lines UACC-62 and Jurkat.

**Compound *^a^***	% Proliferation versus control	
	UACC-62 (melanoma)	Jurkat (lymphoma)
****1****	105 ± 16	88 ± 9
****2****	117 ± 28	107 ± 9
****3****	104 ± 32	92 ± 9
****4****	99 ± 14	128 ± 22
****5****	84 ± 17	41 ± 18
****6****	106 ± 16	110 ± 10
****7****	106 ± 25	93 ± 5
****8****	39 ± 14	32 ± 5
****9****	110 ± 15	99 ± 6
****10****	87 ± 13	113 ± 19
****11****	25 ± 6	120 ± 14
****12****	60 ± 4	54 ± 12
****Cell control****	100	100
****Etoposide****	70 ± 3	60 ± 15

^a^ Compounds **1**-**12** were tested at 100 μM, etoposide was tested at 20 μM. Data shown were means ± S.D of three independent experiments performed in triplicate.

**Figure 2:**
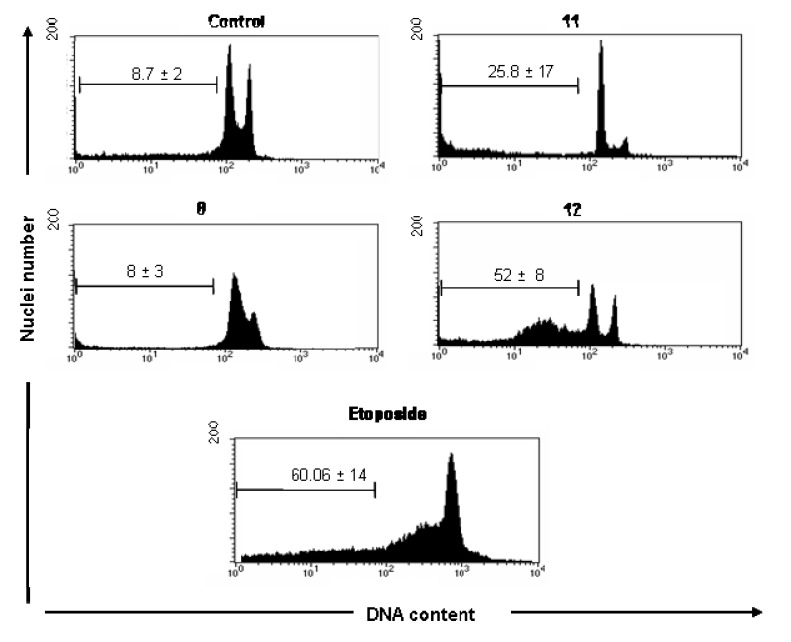
Cell cycle analysis of UACC-62 cells in the absence (control, DMSO 0.5%) and presence of 100 μM of **8, 11 and 12** compounds. Logarithmic representations of fluorescence intensity obtained by PI-staining to distinguish live from apoptotic cells. Sub-G1 peaks are clearly evident after **11** and **12** treatments. Pro-apoptotic drugs, etoposide and camptothencin were used as positive control. Representative data (mean ± SD) of three experiments performed in triplicate. * Statistically different of untreated cell control (p < 0.05).

A significant increase of subdiploid DNA content in UACC-62 cells was observed following treatment with compounds **11** (25.8 ± 17) and **12** (52 ± 8), assayed by increase of sub-G1 peak (Figure 2) when compared with cell control (8.7 ± 2% of cells). Although the compound **8** significantly inhibited the UACC-62 cell proliferation (Table 1), it showed no significantly effect on induction of DNA fragmentation measuring the DNA subdiploid content (8 ± 4), suggesting that the cytotoxic effect of this compound probable involves other mechanism different of apoptosis. In these cells, as expected, etoposide significantly induced apoptosis (49 ± 14) after 18 h of culture.

**Figure 3:**
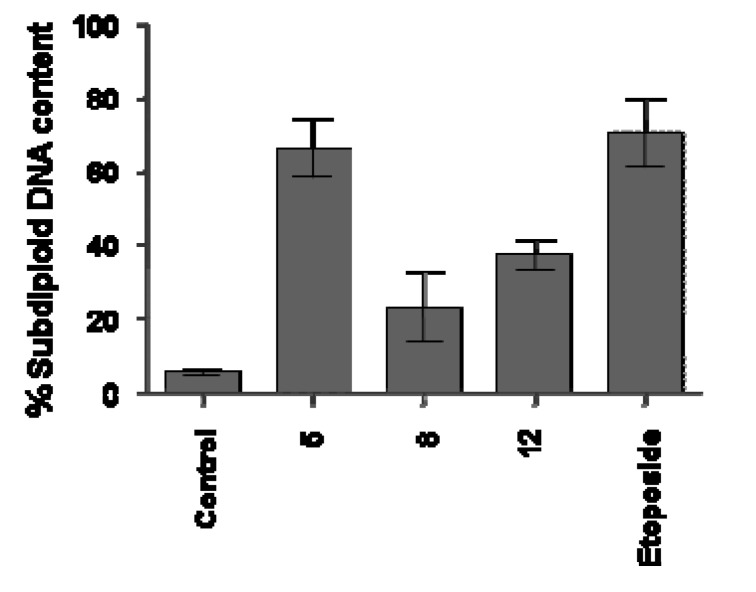
Flow cytometry analyses of DNA content of Jurkat cells treated with synthetic compounds **5, 8** and **12** for 18h. Cells were analyzed with the FACScan flow cytometer as described in the Experimental section.

Different results were observed with Jurkat cells. Compounds **5, 8** and **12** that significantly reduced the cell proliferation (Table 1) demonstrated different impact on DNA fragmentation induction on this line. As shown in Figure 3, the data provided strong evidence that the reduction of proliferation in Jurkat cells after treatment with **8,** as previously verified to UACC-62 cells, is not connected with apoptosis induction, as demonstrated by reduced sub diploid DNA content, after 18 h of treatment with this compound. However, the data clearly showed that compounds **5** and **12**, demonstrated a significant induction of DNA fragmentation (66 ± 17.4% and 37.4 ± 5.9%, respectively) compared with the cell control (5.5 ± 1.9%). In these experiments, etoposide (73 ± 7%) used as the reference substance and known for its proapoptotic behavior, induced DNA fragmentation in a significantly way, as previously described for this chemotherapeutic agent used in clinic.

**Figure 4:**
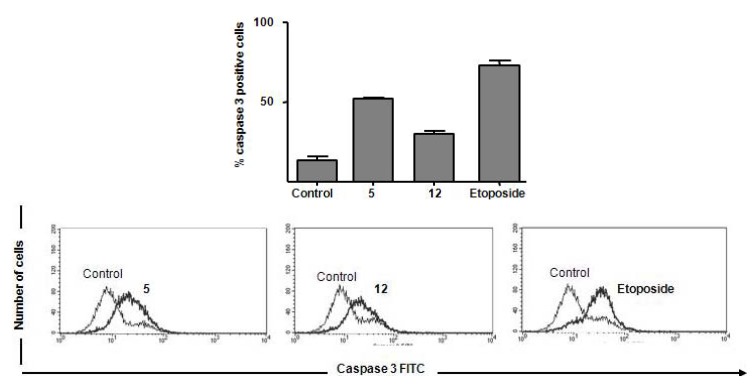
Impact of compounds **5** and **12** on caspase-3 activation. Jurkat cells were treated with compounds for 18 hours and labeled with anti-caspase 3 FITC, following flow cytometric analysis.

To gain insights into the mechanism by which the cytotoxic compounds induce apoptosis, we also investigated their effects on caspases. We focused on caspase-3, which is activated by a number of apoptotic signals. This enzyme is a main executor of apoptosis playing a central role in its biological processing and has been reported that activation of caspase-3 is an essential event for the induction of oligonucleosomal DNA fragmentation [25]. Compounds **5**, **8** and **12** induced an increase in the amount of subdiploid DNA, indicating internucleosomal DNA breakdown, as previously shown (Figure 3). Using the 50th percentile greater values of DNA fragmentation induction by actives substances as a cut-off point, we investigated the substances **5** and **12** on caspase-3 activation in Jurkat cells. Flow cytometry measurements were corroborated by activation of caspase-3 observed in **5** and **12**-treated Jurkat cells (Figure 4). Substance **5** showed additional pro-apoptotic potential when compared with **12** (52 ± 1.3% of caspase-3 positive cells versus 30 ± 2.5%, respectively). Etoposide induced 76 ± 5.8% of caspase activation in Jurkat cells. Cell control (12 ± 1.5%) is represented. Etoposide exerts its antineoplastic activity by inhibiting topoisomerase II which leads to DNA strand breaks, inhibition of DNA replication, and apoptotic cell death.

One of mechanisms involved on apoptotic mechanism induced by etoposide involves the release of mitochondrial cytochrome c leading to the activation of caspase-9. Caspase-9 triggers the activation of caspase-3 [25,26]. Caspase 3 is the major death executioner that orchestrate the dismantling of diverse cell structures through cleavage of specific substrates including the cleavage of ICAD (inhibitor of caspase-activated DNase) releases CAD (caspase-activated DNase), which can then catalyze inter-nucleosomal DNA cleavage [27]. Since oligonucleosomal DNA fragmentation requires activation of caspase-3 [28], it seems reasonable to consider that the oligonucleosomal DNA fragmentation observed on Jurkat cells after treatment with substances **5** and **12** is connected with caspase-3 activation consequently with their pro-apoptotic potential.

Our results demonstrated that **5** and **12** have a pro-apoptotic profile at higher concentrations (100 µM). Langer and co-workers [29] confirmed that owing to the expectation of weak potency of the primary hits, it is necessary to perform biochemical assays of the low molecular weight compounds at relatively high concentrations. Moreover, an actual sample of the presumed hit structure must demonstrate activity in a primary biological assay and an important aspect of selecting chemical series for follow-up is their binding mechanism. In this context, the substances related in the present work can be useful as hit compound to the development of leads to de development of new anticancer drugs. Experiments to determine the IC50 values, as well evaluation of the pro-apoptotic mechanism induced and by compounds and their impact on normal cell lines are currently under investigation in our laboratory.

## Experimental

### General

All melting points were determined on a Kofler Sybron apparatus and are uncorrected. Optical rotations were determined at 25 °C with a Bellingham and Stanley P20 Polarimeter. The IR spectra were recorded on a Shimadsu IR-408 spectrometer. The NMR spectra were recorded on a Bruker *AVANCE* DRX200 or a Bruker *AVANCE* DRX400 instruments, using TMS as the internal standard. Chemical shifts are given in δ (ppm) scale and *J* values are given in Hz. Column chromatography was performed with silica gel 60, 70-230 mesh (Merck). All reagents used were of analytical grade.

### General procedure for synthesis of compounds ***1*** and ***2*** *[13]*

To a solution of **5** [18,19] or **6** [19] (2.0 mmol) in DMF (10 mL) was added *N,N’*-dicyclo-hexylcarbodiimide (2.0 mmol). After 15 minutes at room temperature, a solution of amine **13** [16] (1.0 mmol) in DMF (7 mL) was added, and the stirring was continued for 24 h. The dicyclohexylurea was removed by filtration. The DMF was removed under vacuum and the residue was purified by chromatography using silica gel as the solid support and then eluted with EtOAc/MeOH.

*Methyl 6-(4-bromomethyl-3-nitrobenzoylamino)-6-deoxy-α-D-glucopyranoside* (**1**) [13]: Yield 40%; Mp 140-43 °C; IR (KBr) cm^-1^: 3500-3250 (OH; NH); 1638 (C=O); 1530 (ArNO_2_,N=O assym.), 1331 (ArNO_2_, N=O sym.); 1061,1020 (C-O); ^1^H-NMR (200 MHz, CD_3_OD) δ: 8.50 (d, 1H, *J*_b,f_ = 1.6 Hz, H-b), 8.15 (dd, 1H, *J*_f,b_ = 1.6 Hz, *J*_f,e_ = 8.0 Hz, H-f), 7.83 (d, 1H, *J*_e,f_ = 8.0 Hz, H-e), 5.04 (s, 2H, -C*H*_2_Br), 4.68 (d, 1H, *J*_1,2_ = 3.6 Hz, H-1), 3.86 (s broad, 1H, -O*H*), 3.80 (s broad, 1H, -O*H*), 3.77-3.58 (m, 3H, H-2, H-3 or H-4 and -N*H*), 3.46-3.29 (m, 6H, H-5, H-6 or H-6’, -O*H* and OC*H*_3_), 3.32 (m, 1H, H-4 or H-3), 3.19 (t, 1H, *J*_6,6’_ = 9.0 Hz, H-6’ or H-6); ^13^C-NMR (50 MHz, CD_3_OD) δ: 166.50 (C=O), 148.04 (C-c), 135.63 (C-d), 135.24 (C-a), 131.88 (C-f), 131.68 (C-e), 123.75 (C-b), 99.73 (C-1), 73.29 (C-3), 72.09 (C-2 and C-5), 70.08 (C-4), 54.03 (-O*C*H_3_), 41.54 (C-6), 40.94 (-*C*H_2_Br).

*Methyl 6-(4-acetoxymethyl-3-nitrobenzoylamino)-6-deoxy-α-D-glucopyranoside* (**2**) [13]: Yield 45%; Mp 156.3-157.6 °C; [α]_D_ = +90.3 (*c* 0.6 in methanol); IR (KBr) cm^-1^: 3550-3200 (OH; NH); 1740 (C=O); 1640 (C=O); 1530 (ArNO_2_,N=O assym.), 1325 (ArNO_2_, N=O); 1230 (C-C(=O)-O); 1000-980 (C-O); ^1^H-NMR (400 MHz, CD_3_CN) δ: 8.47 (d, 1H, *J*_b,f_ = 1.6 Hz, H-b), 8.10 (dd, 1H, *J*_f,b_ = 1.6 Hz, *J*_f,e_ = 8.1 Hz, H-f), 7.74 (d, 1H, *J*_e,f_ = 8.1 Hz, H-e), 7.44 (s broad, 1H, N*H*), 5.45 (s, 2H, -C*H*_2_OAc), 4.62 (d, 1H, *J*_1,2_ = 3.7 Hz, H-1), 3.85 (d, 1H, *J* = 4.2 Hz, -O*H*), 3.65-3.58 (m, 3H, H-2, H-3 or H-4 and -O*H*), 3.50-3.45 (m, 1H, H-5), 3.32-3.29 (m, 5H, H-6 or H-6’, -O*H* and –OC*H*_3_), 3.12 (td, 1H, *J* = 8.9 Hz, *J* = 4.2 Hz, H-4 or H-3), 2.87 (d, 1H, *J*_6,6’_ = 7.7 Hz, H-6’ or H-6), 2.11 (s, 3H, -COC*H*_3_); ^13^C-NMR (100 MHz, CD_3_CN) δ: 171.27 (C=O), 166.53 (C=O), 148.46 (C-c), 136.18 (C-d), 136.00 (C-f), 133.16 (C-e), 130.27 (C-a), 124.85 (C-b), 100.84 (C-1), 74.56 (C-3), 73.31 (C-2), 72.57 (C-5), 71.31 (C-4), 63.32 (-*C*H_2_OAc), 55.58 (-O*C*H_3_), 41.83 (C-6), 20.90 (-CO*C*H_3_).

### 4-Bromomethyl-3-nitrobenzamide *(**7**) [13]*

Silica gel (0.38 g, MerK Kieselgel 60, particle size 0.063-0.200 mm, 70-230 mesh) was mixed with a solution of ammonium chloride (0.08 g; 1.5 mmol), in water (5.0 mL). Evaporation of water under reduced pressure gave a dry white powder, which was used as the amine source [21]. Then, the silica-supported ammonium salt was transferred to a pestle and mortar and well-ground with **5** [18,19] (0.20 g; 0.77 mmol) and TsCl (0.15 g; 0.78 mmol). To this mixture was added triethylamine (0.4 g) and mixed by a spatula. After 5 min, the reaction mixture was washed with 0.02 N solution of HCl (2 × 15 mL). The aqueous layer was extracted twice with ethyl acetate (20 mL). The combined organic layers were dried over anhydrous sodium sulfate and the solvent was removed. The crude product was purified by column chromatography on silica gel using silica gel as the solid support and then eluted with EtOAc/hexane (7:3 v/v) mixture [21]. Yield: 16%; Mp: 117.1-118.7 ^o^C; IR (KBr) cm^-1^: 3406, 3178 (N-H),.1653 (C=O), 1531 (ArNO_2_, N=O assym.), 1343 (ArNO_2_, N=O sym.); ^1^H-NMR (200 MHz, DMSO-d6) δ: 8.52 (d, 1H, *J*_b,f_ = 1.4 Hz, H-b), 8.32 (s broad, 1H, N*H*), 8.22 (dd, 1H, *J*_f,b_ = 1.4 Hz and *J*_f,e_ = 8 Hz, H-f), 7.88 (d, 1H, *J*_e,f_ = 8 Hz, H-e), 7.74 (s broad, 1H, N*H*), , 5.08 (s, 2H, C*H*_2_).

### N-Benzyl-4-bromomethylbenzamide *(**8**) [13]*

A solution of 4-(bromomethyl)benzoic acid [19] (0.40 g; 1.86 mmol) in thionyl chloride (2.2 mL) and dry chloroform (2 mL) was stirred under reflux for 3 hours. The solvent was removed and the obtained residue (0.43 g; 1.84 mmol) was dissolved in THF (1 mL) and dichloromethane (1 mL) and added dropwise to a mixture of benzylamine (0.20 mL; 1.86 mmol) and triethylamine (0.29 mL, 2.10 mmol) in dry THF (1 mL) cooled in an ice bath. The reaction mixture was diluted with dichloromethane (2 mL) and stirred at room temperature for 3 h. Crushed ice and 6 mol/L HCl solution were then added to the reaction mixture until pH 1. The resulting solid was filtered and washed with water. Yield: 50%; Mp: 142.9-144.9 °C; IR (KBr) cm^-1^: 3313 (NH); 3059-3029 (Ar-H), 2932 (C-H),1639 (C=O); 1548 (N-H); 1259 (CH_2_-Br); ^1^H-NMR (200 MHz, CDCl_3_) δ: 7.66 (d, 2H, *J*_b,c_ = 8.2 Hz, 2 *×* H-b), 7.31 (d, 2H, *J*_c,b_ = 8.2 Hz, H-c), 7.24 (m, 5H, Ar-H), 6.65 (s broad, 1H, N*H*), 4.51 (d, 2H, *J*_CH2,NH_ = 5.7 Hz, -C*H*_2_NH-), 4.39 (s, 2H, *-*C*H*_2_Br); ^13^C-NMR (50 MHz, CDCl_3_) δ: 166.75 (C=O), 141.14 (C-e), 138.02 (C-d), 134.20 (C-a), 129.14 (2 *×* C-b), 128.70 (2 *×* C-c), 127.78 (2 *×* C-g), 127.54 (C-h), 127.47 (2 *×* C-f), 44.03 (-*C*H_2_NH-), 32.26 (-*C*H_2_Br).

### N-Benzyl-4-benzylaminomethylbenzamide *(**9**) [13]*

A solution of 4-(bromomethyl)benzoic acid [19] (0.47 g; 2.18 mmol) in thionyl chloride (2.5 mL) and dry chloroform (2 mL) was stirred under reflux for 3 hours. The solvent was removed and the obtained residue (0.51 g; 2.18 mmol) was dissolved in dichloromethane (8 mL) and added dropwise to a mixture of benzylamine (1.66 mL; 15.2 mmol) in dry THF (3 mL) cooled in an ice bath. The reaction mixture was stirred at room temperature for 72 h. The reaction mixture was filtered and the excess of benzylamine and the solvent were removed from the filtrate. The residue was dissolved in methanol and to this solution was added a solution of sodium methoxide in methanol (0.2 g in 10 mL). This mixture was stirred at room temperature for 1 h. The methanol was removed and the residue was taken up in water (30 mL) and extracted with ethyl acetate (4 × 50 mL). The combined extracts were dried over anhydrous sodium sulfate, filtered and the solvent was removed under reduced pressure. The oil obtained was purified by column chromatography using silica gel as the solid support and then eluted with ethyl acetate/hexane (8:2 v/v) mixture. Yield: 40%; Mp: 81.1-84.5 ^o^C; IR (KBr) cm^-1^: 3285 (NH); 3066-3031 (Ar-H), 2921, 2856, 2824 (C-H), 1633 (C=O); 1550 (N-H); ^1^H-NMR (200 MHz, DMSO-d6) δ: 9.97 (s broad, 1H, -N*H*), 9.24 (t, 1H, *J*_NH,CH2_ = 5.7 Hz, -N*H*), 7.95 (d, 2H, *J*_b,c_ = 8.1 Hz, 2 *×* H-b), 7.67 (d, 2H, *J*_c,b_ = 8.1 Hz, 2*×* H-c), 7.59-7,21 (m, 10H, Ar-H), 4.48 (d, 2H, *J*_CH2,NH_ = 5.7 Hz, -C*H*_2_NH-), 4.19-4.13 (m, 4H, 2 *×* -C*H*_2_NH-); ^13^C-NMR (50 MHz, DMSO-d6) δ: 165.70 (C=O), 139.65 (C-d), 134.99 (C-e or C-i), 134.58 (C-i or C-e), 131.86 (C-a), 130.25 (2 *×* C-b), 130.13 (2 × C-c), 128.93 (C-h), 128.60 (2 *×* C-g or 2 *×* C-k), 128.30 (2 *×* C-k or 2 *×* C-g), 127.48 (2 *×* C-j or 2 *×* C-f), 127.26 (2 *×* C-f or 2 *×* C-j), 126.77 (C-l), 50.18 (-*C*H_2_NH-), 49.67 (-*C*H_2_NH-), 43.01 (-*C*H_2_NH-).

### 2,5-Bis-(3,4,5-trimethoxyphenyl)-3-formylfuran *(**12**)*

Under ice-bath cooling, 5 drops of POCl_3_ were added to a solution of **14** [22] (0.18 g; 0.44 mmol) in 1,2-dichloroethane (3 mL) and DMF (2 mL). The mixture was then heated under reflux for 48 h. After cooling to room temperature, the reaction mixture was diluted with water (50 mL), washed with diethyl ether (3 *×* 30 mL). The combined extracts were then dried over anhydrous sodium sulfate and the solvent was removed. Unreacted starting material was recovered from the residue obtained. During this period, a solid precipitates from aqueous phase. The solid was separated by filtration and characterized as being the product **12**. Yield: 11%; Mp: 110-115 °C; ^1^H-NMR (200 MHz, DMSO-d6) δ: 10.19 (s, 1H, -C*H*O), 7.12 (d, 2H, *J*_b,b’_ = 3.5 Hz, H-b and H-b’), 7.02 (s, 1H, H-4), 6.97 (d, 2H, *J*_f,f’_ = 3.5 Hz, H-f and H-f’), 3.97 (s, 3H, OC*H*_3_), 3.88 (s, 3H, OC*H*_3_), 3.86 (s, 6H, 2*×*OC*H*_3_), 3.82 (s, 3H, OC*H*_3_), 3.69 (s, 3H, OC*H*_3_). ESI-MS [M + H]^+^ 429, [M+Na]^+^: 451.

### Biological evaluation

RPMI-1640 and L-glutamine were obtained from GIBCO (Grand Island, NY, USA). Heat-inactivated, calf serum were obtained from Flow Laboratories (Royaune, UK). MTT, propidium iodide, Etoposide and CBolchicines were purchased by Sigma (St Louis, MA, USA), anti-caspase-3 FITC (BD Pharmingen, San Jose, CA, USA).

Human cancer cell lines UACC-62 was purchased from the National Cancer Institute (Bethesda, MD, USA) and Jurkat cells were generously provided by Dr. Amarante-Mendes (University of São Paulo). All cells were maintained in RPMI 1640 supplemented with 2 mM L-glutamine, gentamicin (50 μg/mL) and 5% or 10% of inactivated foetal bovine serum for UACC-62 or Jurkat cells, respectively. Cells were incubated at 37 °C in a humidified, 5% CO2 atmosphere.

#### Antitumor activity

The assay with the human cancer cell lines UACC-62 (human melanoma) and Jurkat (human leukemia) was run using the protocol established at the National Cancer Institute [30] with modifications. In brief, UACC-62 cells are detached from the culture flaks by addition 1 mL of 0.05% trypsin-EDTA (GIBCO). After counting, dilutions are made to give appropriated cell densities for inoculating onto the microtiter plates. UACC-62 cells are inoculated in a volume of 100 μL per well at densities of 10,000 cells per well and are preincubated for 24 hours at 37 °C to allow stabilization prior to addition of substances. This pre-incubation procedure was performed to Jurkat cells (50.000 cels/well) prior of treatment with the substances. Both lineages were incubated with substances at 100 μM for 48 hours and the cell proliferation/viability measured by MTT method as previously described [23]. In all experiments, etoposide (20 μM) was the positive control of chemotherapeutic used in clinic. Cell treated only with the solvent (DMSO at 0.5%) as used as control. The results are expressed as the average of triplicate assays.

#### Analysis of DNA Fragmentation - DNA labeling and flow cytometry analysis

We selected cytotoxic substances to one or both lineages and evaluated their pro-apoptotic potential. We used the method to detect apoptotic nuclei, as described by Nicoletti and colleagues [24]. The UACC-62 (5 *×* 10^4^ cell/well) and Jurkat cells (2 *×* 10^5^ cells/well) were treated or not with cytotoxic substances for 18h, and where centrifuged and resuspended in hypotonic solution (50 μg/mL PI in 0.1% sodium citrate plus 0.1% Triton X-100). The samples were incubated 4h at 4 °C, and PI fluorescence of individual nuclei was measured using a FACScalibur flow cytometer (Becton Dickinson Immunocytometry Systems, San Jose, CA, USA). The data were analyzed using the Lysis software (Becton Dickinson). The percentage of hypodiploid nuclei correlates with the extent of apoptosis in the samples. The results represent the average ± SD in triplicate samples. Every experiment was repeated at least three times.

#### Flow cytometric analysis of caspase 3 activation

The percentage of Caspase 3 active in Jurkat cells exposed to substances was determined by a single staining with a Caspase 3-FITC Antibody (BD Biosciences, San Jose, CA) and using flow cytometric analysis (FACSCalibur and Cell Quest Pro Software, BD Biosciences). Cells (4 *×* 10^5^) were washed twice with PBS, resuspended in 50 μL of 1× binding buffer and incubated on ice for 20 minutes. Cells were then washed twice if Perm/Wash buffer (0.5 mL). Cells were then resuspended in Perm/Wash buffer and antibody for thirty minutes at room temperature. The cells were then washed and re-suspended in binding buffer and flow cytometric analysis were performed within 30 minutes of staining using FL1 versus FL2 dot plot analysis acquiring 10,000 events. The instrument settings were optimized using a negative control of untreated Jurkat cells and a positive control of etoposide treated Jurkat cells. Results were analyzed and statistical analysis done using the Cell Quest Pro software BD Biosciences.

## 4. Conclusions

Twelve synthetic compounds were tested as potential cytotoxic agents against two cancer cell lines: melanoma (UACC-62) and lymphoma (JURKAT). Primary screens were performed at a final compound concentration of 100 μM. Among the twelve, four were found to be effective against one or both of the cancer cells lines. Compounds **5**, **8**, **11** and **12**, which showed cytotoxicity, were evaluated in an apoptosis assay. Compounds **5** and **12** showed a good apoptotic response against Jurkat cell leukemia culture whereas the **8** and **11** had minor impact on induction of DNA fragmentation, thereby suggesting the existence of different mechanisms of action. These compounds displayed promising activity and could be used as leads in the design and development of new anticancer drugs. A structure-activity relationship cannot be established due to the structural variation of the compounds examined in this exploratory work.
